# IFI27-mediated regulation of regulatory T cells aggravates lung injury in sepsis via IL-10/STAT3 signaling

**DOI:** 10.3389/fimmu.2026.1760728

**Published:** 2026-04-22

**Authors:** Juan Li, Yingying Huang, Chaoping Ma, Guoxiang Liu, Caiting Chu, Jiameng Chen, Huiqi Wang, Jiawei Ye, Min Jiao, Yiran Guo, Jiyuan Zhang, Yuxin Leng, Chengjin Gao

**Affiliations:** 1 Department of Emergency, Xinhua Hospital, Shanghai Jiao Tong University School of Medicine, Shanghai, China; 2 Dementia Research Centre, Department of Biomedical Sciences, Faculty of Medicine Health and Human Sciences, Macquarie University, Sydney, NSW, Australia; 3 Department of Radiology, Xinhua Hospital, Shanghai Jiao Tong University School of Medicine, Shanghai, China; 4 Department of Intensive Care Unit, Peking University Third Hospital, Beijing, China

**Keywords:** IFI27, IL-10, regulatory T cells, sepsis, STAT3

## Abstract

**Background and purpose:**

Interferon-induced protein 27 (IFI27) is implicated in immune regulation, and regulatory T cells (Tregs) play a critical role in maintaining pulmonary immune homeostasis during sepsis. However, the relationship between IFI27 and Treg-mediated regulation in sepsis-associated lung injury remains unclear. This study aimed to investigate the role of IFI27 in modulating Treg function during sepsis.

**Methods:**

Plasma IFI27 levels were measured in patients with sepsis and healthy controls. mRNA sequencing was performed to assess IFI27 expression profiles. *In vivo*, IFI27 expression was examined in a cecal ligation and puncture (CLP) mouse model, and an IFI27 overexpression mouse model was used to evaluate its functional role in sepsis. Immunofluorescence staining and transmission electron microscopy were employed to assess ferroptosis in lung epithelial cells. Flow cytometry was used to analyze Treg populations and their secretion of interleukin-10 (IL-10). *In vitro*, primary Tregs were co-cultured with mouse lung epithelial cells to determine the effects of IFI27 on IL-10 secretion in Tregs and epithelial ferroptosis. Reactive oxygen species (ROS) levels, malondialdehyde (MDA) content, and STAT3 pathway-related protein expression were quantified using ROS assays, MDA measurements, and western blotting, respectively.

**Results:**

Clinical analyses demonstrated that plasma IFI27 levels were positively correlated with sepsis severity. IFI27 expression was significantly increased in septic mice. Elevated IFI27 inhibited STAT5 phosphorylation, leading to a reduction in Treg abundance and IL-10 secretion, thereby exacerbating ferroptosis in pulmonary epithelial cells. Furthermore, IFI27 elevation in Tregs increased lipid peroxidation levels by suppressing the IL-10/STAT3 signaling pathway.

**Conclusions:**

These findings indicate that IFI27 is closely associated with sepsis severity and may serve as a potential prognostic indicator. Mechanistically, IFI27 suppresses Treg function and enhances ferroptosis in lung epithelial cells through inhibition of the IL-10/STAT3 signaling pathway, thereby aggravating sepsis-induced lung injury.

## Introduction

Sepsis is a life-threatening organ dysfunction syndrome caused by a dysregulated host response to infection and remains one of the leading causes of mortality worldwide (1). Sepsis frequently results in dysfunction of multiple organs, including the lungs, heart, liver, and kidneys, with the lungs being the most commonly and severely affected organ (2). In sepsis-induced lung injury, immune cell infiltration represents a major pathogenic contributor (3). The overwhelming inflammatory response characteristic of sepsis-associated lung injury is orchestrated by both innate and adaptive immune cells (4). Innate immune cells, including neutrophils, macrophages, and natural killer T cells, play pivotal roles in initiating and amplifying the systemic inflammatory cascade that drives lung injury during sepsis (5–7). In addition, regulatory T cells (Tregs) have been shown to exert direct immunomodulatory effects on the innate immune response in sepsis-induced lung injury (8).

Epithelial injury is a central pathological feature of sepsis-induced acute respiratory distress syndrome (ARDS) (9). Multiple immune cell populations and signaling pathways participate in both the injury and repair of lung epithelial cells. Among these, Foxp3^+^ Tregs facilitate lung injury repair by modulating immune responses and promoting alveolar epithelial cell proliferation (10–12). During sepsis-induced ARDS, excessive inflammatory responses lead to severe lung injury, whereas Tregs counteract this process by secreting anti-inflammatory mediators such as interleukin-10 (IL-10) and transforming growth factor- β (TGF-β), thereby limiting inflammation and protecting lung epithelial cells from inflammatory damage (13, 14). Although Treg-derived IL-10 is known to regulate lung epithelial cell function, the precise regulatory mechanisms within epithelial cells remain insufficiently characterized and require further investigation. In ARDS both epithelial barrier integrity and alveolar fluid clearance capacity are markedly impaired (15). Circulating factors, including damage-associated molecular patterns, microbial products, immune cells, and inflammatory mediators, contribute to epithelial cell injury (16). Ferroptosis, a regulated form of cell death driven by iron accumulation and reactive oxygen species (ROS), has recently been recognized as an important contributor to the progression of sepsis-induced ARDS. Clinical studies have reported pronounced alterations in iron metabolism and elevated lipid peroxidation levels in lung tissues from patients with sepsis-induced ARDS, implicating ferroptosis in the exacerbation of lung injury (17).

Interferon-induced protein 27 (IFI27) is a canonical interferon-stimulated gene that encodes a protein increasingly recognized as a regulatory node within inflammatory and immune signaling networks (18). IFI27 has been identified as a genetic risk factor in sepsis-induced ARDS, primary Sjogren’s syndrome, and systemic lupus erythematosus (SLE) (19–21). These findings suggest that IFI27 plays a critical role in regulating inflammatory signaling and immune homeostasis (22, 23). However, the functional contribution of IFI27 to the immunopathogenesis of sepsis remains poorly understood. IFI27 has been shown to regulate interferon and JAK–STAT signaling pathways in neutrophils, memory B cells, and CD8^+^ T cells, thereby contributing to immune dysregulation in SLE (24). In studies of COVID-19 and idiopathic inflammatory myopathy (IIM), IFI27 was identified as a shared differentially expressed gene and demonstrated a positive association with M1 macrophage (25). Moreover, recent evidence indicates that IFI27 can inhibit Treg infiltration (26). Given that Tregs are critical modulators of immune homeostasis and serve to restrain excessive inflammation during sepsis-induced lung injury (8, 27), these findings suggest a potential role for IFI27 in suppressing Treg-mediated immune regulation. Nevertheless, the specific role of IFI27 in regulating Treg function during sepsis has not been systematically investigated.

In the present study, we examined the role of IFI27 in sepsis pathogenesis with a particular focus on its regulation of Treg function. Using an IFI27 overexpression mouse model, we observed that elevated IFI27 expression aggravated tissue injury and was associated with a reduction in Treg abundance. Furthermore, Tregs from IFI27-overexpressing mice exhibited diminished IL-10 secretion compared with those from wild-type controls. The reduction in IL-10 production was accompanied by enhanced ferroptosis in alveolar epithelial cells. Collectively, these findings indicate that IFI27 contributes to the pathogenesis of sepsis by impairing the immunosuppressive function of Tregs, thereby exacerbating lung epithelial injury.

## Methods

### Patient samples

Peripheral blood samples were collected from 58 patients with sepsis and 53 non-septic control subjects at Xinhua Hospital affiliated with Shanghai Jiao Tong University School of Medicine. Among these samples, RNA extracted from the peripheral blood of four patients with sepsis and four controls was used for transcriptomic sequencing. All sample collection procedures were approved by the institutional ethics committee (Approval No. XHEC-D-2024-023). The researchers strictly followed the Declaration of Helsinki and fully respect the subjects’ rights to know and privacy, and effectively protect the subjects’ rights and well-being. Our studies were reviewed and approved by Ethics Committee at the Xinhua Hospital affiliated to Shanghai Jiao Tong University School of Medicine. Sepsis was diagnosed according to the Third International Consensus Definitions for Sepsis and Septic Shock (Sepsis-3.0). Sepsis is defined as the presence of confirmed or suspected infection, together with a sequential Sepsis-related Organ Failure Assessment (SOFA) score increase of≥2 points, assessed by trained doctors following hospital admission. Non-septic controls were defined as hospitalized patients without infection and with a Sequential Organ Failure Assessment (SOFA) score of less than 2 points. Exclusion criteria included (1): age under 18 years; (2) pregnancy or breastfeeding; (3) human immunodeficiency virus infection or severe immunosuppression; (4) receipt of chemoradiotherapy, radiotherapy, or corticosteroid treatment within 30 days prior to enrollment; and (5) inability to provide informed consent; (6) the primary infectious focus was located in the lungs.

### RNA sequencing and analysis

Total RNA was isolated using TRIzol reagent (Thermo Fisher Scientific, Waltham, MA, USA) according to the manufacturer’s instructions. RNA libraries were constructed using an Illumina HiSeq platform and sequenced on a DNBSEQ-T7 gene sequencer. Downstream bioinformatic analyses were performed using standard pipelines, and functional enrichment analysis was conducted using the *clusterProfiler* R package.

### Animals

All mice used in this study were male C57BL/6J mice aged 6–8 weeks and weighing 20–25 g. Animals were randomly assigned to experimental groups. All procedures were performed in accordance with the National Institutes of Health guidelines for the care and use of laboratory animals and were approved by the Institutional Animal Care and Use Committee of Xinhua Hospital (Approval No. XHEC-D-2024-023).

### Cecal ligation and puncture model and AAV-IFI27 construction

The CLP model was established as previously described (15). Briefly, mice were anesthetized by intraperitoneal injection of 3% pentobarbital sodium at a dose of 30 mg/kg prior to surgery. To generate IFI27-overexpressing mice, adeno-associated virus (AAV) vectors carrying a construct conferring IFI27 overexpression were administered via intranasal delivery. IFI27 knockout mice were generated via the homologous recombination method and maintained under specific pathogen-free conditions, with a 12-hour light/12-hour dark cycle and a controlled ambient temperature of 22–24°C. The targeting strategy of the IFI27 knockout mice was shown in **Supplementary Figure 3**. Finally, CO2 was introduced at a displacement rate of 30–70% of the chamber volume per minute and at a steady flow rate to rapidly induce loss of consciousness, thereby minimizing distress as much as possible. A 10−liter euthanasia chamber was used with a flow rate maintained at 5 liters per minute.

### Histological analysis

Mouse lung tissues were fixed in 4% paraformaldehyde (Sigma-Aldrich, St. Louis, MO, USA) for more than 48 h, embedded in paraffin, and sectioned into 5-µm-thick slices. Sections were stained with hematoxylin and eosin (H&E). Lung injury was evaluated using the Smith pathological scoring system, which grades pulmonary edema, alveolar and interstitial inflammation, alveolar and interstitial hemorrhage, atelectasis, and hyaline membrane formation on a scale of 0–4.

### Lung wet-to-dry weight ratio

Pulmonary edema was assessed by measuring the lung wet-to-dry weight ratio. Lung tissues were weighed immediately after excision to obtain wet weight and then dried at 65°C for 72 h to determine dry weight. The W/D ratio was calculated as wet weight divided by the dry weight.

### RNA isolation and quantitative real-time PCR

Total RNA from lung tissues and cultured cells was extracted using TRIzol reagent (Thermo Fisher Scientific). Complementary DNA was synthesized, and quantitative real-time PCR was performed using standard protocols. Gene expression levels were normalized to those of GAPDH and calculated using the 2^−ΔΔCt^ method. Primer sequences used for amplification are listed in **Supplementary Table 1**.

### Cytokine ELISA

Levels of IL-6, IL-1β, TNF-α, and IL-10 in mouse serum and lung tissue homogenates were quantified using commercially available ELISA kits (Cusabio) in accordance with the manufacturer’s instructions.

### Immunohistochemistry

Paraffin-embedded lung tissue sections were deparaffinized and rehydrated, followed by antigen retrieval at 95°C for 30 min. Sections were incubated overnight at 4°C with rabbit anti-IFI27 primary antibody (1:150, Novus, USA) in a humidified chamber. After incubation with secondary antibodies at room temperature for 30 min, images were captured using an Olympus microscope equipped with an Olympus camera(Japan).

### Immunofluorescence

After dewaxing and dehydrating of lung tissue sections, 5% bovine serum albumin was used to block each slide for 30min and then incubate with primary antibody overnight at 4°C in a humidified black chamber and secondary antibody at room temperature for 1h. Fluorescence intensity analysis was performed using ImageJ software 1.8.0 (Bethesda, USA). Primary antibodies used were anti-GPX4(1:200, CST, USA), anti-FSP1(1:500, CST, USA).

### Transmission electron microscopy

Mouse fresh lung tissue (not exceeding 1mm×1mm×1mm in volume) fixed in glutaraldehyde (Beyotime, China) at 4°C for 2–4 hours, and then applied 1% osmic acid and 0.1M phosphate buffer PBS to fix again at room temperature for another 2h. Then the samples were dehydrated, infiltrated, embedded and cut into ultrathin sections of 40-50nm. The slices were stained with uranium and lead (2% uranium acetate saturated solution, lead citrate, staining for 15min each), and dried overnight at room temperature. The images of sections were taken by transmission electron microscope (FEI, Netherlands).

### Flow cytometry

Single cell suspensions were stained with the following monoclonal antibodies with fluorescence: L/D FVS510 (BD Pharmigen), CD45 APC-CY7 (BD Pharmigen), CD3 PE-CF594 (BD Pharmigen), CD4 FITC (BD Pharmigen), CD8 Percp-CY5.5 (BD Pharmigen), Foxp3 APC (BD Pharmigen), IL-10 BV605 (BD Pharmigen). Cells were first stained with surface antibodies and then fixed and permeabilized with intracellular fixation and permeabilization buffer kit (eBioscience, USA). For intracellular IL-10 staining, cells were first incubated with leukocyte activation cocktail (BD, GolgiPlug™) for 4–6 hours, then stained with surface and intranuclear antibodies. Data were acquired using BD LSRFortessa X20 cell analyzer (BD Biosciences, San Jose, CA, USA) and analyzed with FlowJo 10.0 software.

### BEAS-2B cell culture and Lenti-IFI27 establishment

Human lung epithelial cells (BEAS-2B) were cultured in DMEM medium (Gibco, USA) supplemented with 10%FBS (Gibco, USA) and 1% penicillin-streptomycin (Gibco, USA). BEAS-2B cells were plated in 6-well plate to a density of 90-95% before transfection and then prepare the DNA-Hieff Trans™ Liposome Nucleic Acid Transfection Reagent Complex. Mix the diluted DNA and the diluted liposomal nucleic acid transfection reagent and incubate at room temperature for 20minutes and subsequently add 500uL of the complex to each well of the cell culture plate incubating at 37°C in a 5%CO_2_ incubator for 48h. Cell pellets were collected and isolated using Trizol reagent for mRNA-Sequencing.

### Depletion of Tregs

To deplete regulatory T cells (Treg cells), PC61 mAb (anti-murine CD25 rat IgG1) was injected intraperitoneally (ip) at a dosage of 200ug/mouse. This treatment was initiated 3 days before CLP surgery.

### Co-culture of Tregs and lung epithelial cells

Lung epithelial cells (TC-1) were cultured in RPMI 1640 medium (Gibco, USA) supplemented with 10%FBS (Gibco, USA) and 1% penicillin-streptomycin (Gibco, USA). Tregs were isolated from the spleen of mice using the EasySep™ Mouse CD4+CD25+ Regulatory T Cell Isolation Kit II (stemcell, Vancouver, Canada). The primary Tregs were cultured in RPMI 1640 medium and were stimulated with anti-CD3(1ug/mL), anti-CD28(1ug/mL), IL-2(10ng/mL). The culture medium was changed every 2 days, antibodies and stimulating factors anti-CD28 (1ug/mL), IL-2 (10 ng/mL) were supplemented and continued stimulation for another 2–3 days. Once the Tregs proliferated to a sufficient quantity, the culture medium was removed from the TC-1 cells, and Tregs were added for co-culture in 24-well plates. Adherent TC-1 cells and suspension Treg cells were directly cultured together at a ratio of 1:2 (Treg: TC-1). After 24 hours of co-culture, the cells were treated with LPS (2 μg/mL, Sigma), followed by the addition of IL-10 (10 ng/mL, Proteintech) at 48 hours. Samples were collected for subsequent analyses 24 hours after IL-10 stimulation.

### Cell death detection

Annexin V-FITC Detection Kit was applied to detect cell death. Harvest cells were incubated with 5 μl Annexin V-FITC and 10 μl PI for 10-20min in the dark, and then analyzed with Beckman CytoFlex S cell analyzer (Beckman Coulter, USA). Cells positive for both Annexin V-FITC and PI were considered as dead cells.

### MDA measurement

Cells were cleaved using protein lysate and centrifuged at 12000 g for 10 min. Samples, standards and MDA detection working fluid were added into each tube, and then incubate the mixture in 100°C for 15 min. Then centrifuged at 1000g for 10 minutes. Added 200 μL supernatant into a 96-well plate, and then the absorbance of 532nm was measured and calculated.

### Western blotting

Primary Treg cells and TC-1 cells protein were collected using RIPA lysis buffer with protease and phosphatase inhibitors (Beyotime, China). After electrophoresis, the proteins were transferred onto a PVDF membrane. After blocking with 5% non-fat milk at room temperature for 2 hours, the membranes were incubated with primary antibodies as follows: anti-IFI27(Protein-tech, China, 1:1000), anti-STAT5 (Abcam, USA, 1/500 - 1/2000), anti-STAT5(phospho Y694) (Abcam, USA, 1/1000-1/10000), anti-STAT3 (Abcam, USA, 1/1000-1/2000), anti-STAT3(Tyr705) (Abcam, USA, 1/2000-1/20000), β-actin (Abcam, USA, 1:1000). After being incubated at 4°C overnight, the blots were then incubated with the second anti-rabbit antibody (Abcam, USA, 1/2000 - 1/20000). Signals were ultimately detected using the ECL method. The western blot bands were quantified using Image J (V l.8.0).

### Statistical analysis

Normally distributed continuous variables were presented as mean ± standard deviation (SD) and were compared using Student’s t-test or analysis of variance (ANOVA). Continuous variables with a non-normal distribution were summarized as median with interquartile range (25th–75th percentiles) and were analyzed using the Mann-Whitney U test or Kruskal-Wallis test. Categorical data were expressed as frequency and percentage. Associations between two variables were assessed using the nonparametric Spearman’s rank correlation coefficient. Each experiment was conducted in triplicate. Data shown are representative of three independent experiments. All statistical analyses were conducted using SPSS software (version 19.0; IBM Corp.) and GraphPad Prism (version 5.01; GraphPad Software, Inc.). P value less than 0.05 was considered statistically significant.

## Results

### IFI27 is increased in sepsis and associated with disease severity in septic patients

**Figure 1:**
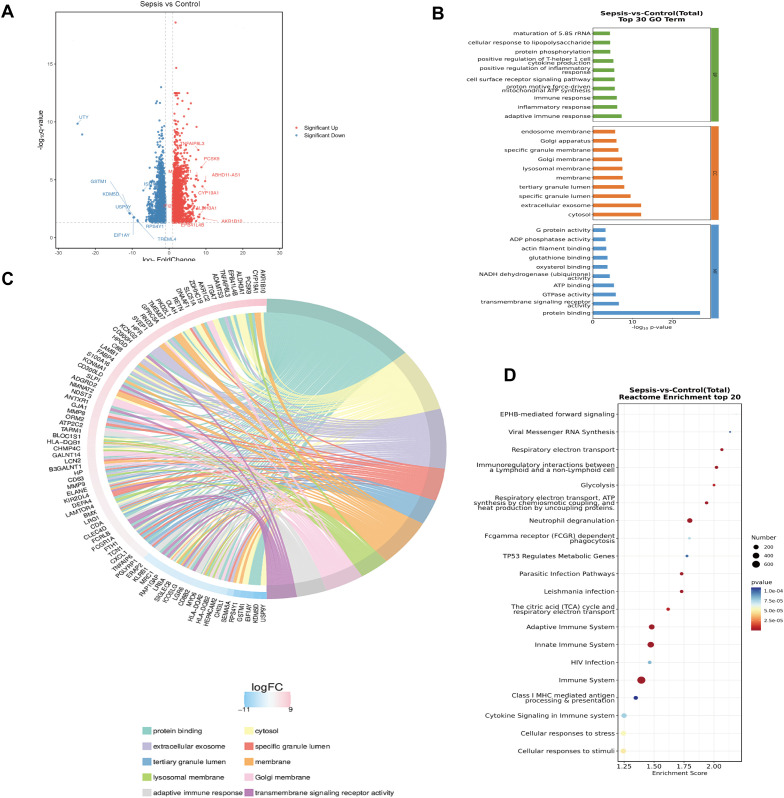
IFI27 was increased in sepsis. **(A)** Volcano plot of differentially expressed genes in septic patients. The top upregulated ten genes were ABHD11-AS1、AKR1B10、CYP19A1、PCSK9、ALDH3A1、EPB41L4B、TNFAIP8L3、IFI27、MAFA-AS1、STC2. The top ten downregulated genes were ISM1、DDX3Y、ZFY、TREML4、RPS4Y1、GSTM1、EIF1AY、KDM5D、USP9Y、UTY. **(B, C)** Enriched gene ontology (GO) function analysis and Chord diagram of upregulated genes. **(D)** Kyoto Encyclopedia of Genes and Genomes (KEGG) enrichment analysis and Chord diagram of upregulated genes.

**Figure 2:**
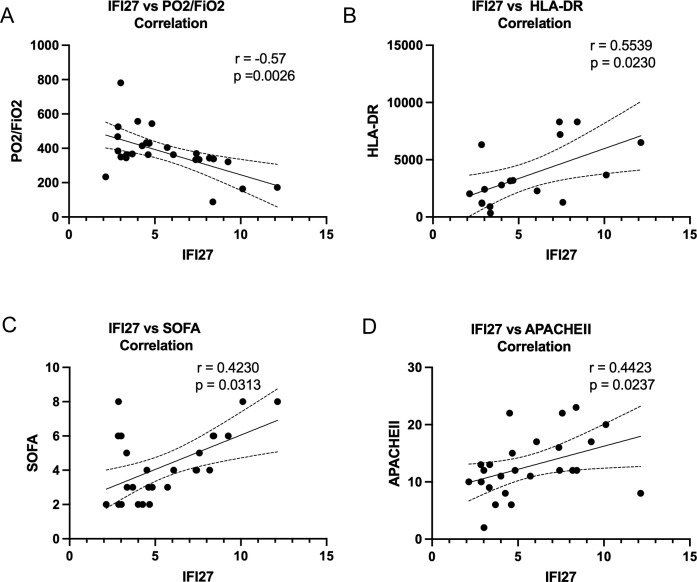
IFI27 was associated with the severity of sepsis. **(A-D)** Correlation of the expression of IFI27 mRNA with PaO2/FiO2 ratio, SOFA score, APACHEII and HLA-DR in patients with sepsis respectively. Dots represent each individual participant. P values less than 0.05 were considered statistically significant.

Differential expression analysis of transcriptomic sequencing data from four patients with sepsis and four controls identified the top ten upregulated genes as ABHD11-AS1, AKR1B10, CYP19A1, PCSK9, ALDH3A1, EPB41L4B, TNFAIP8L3, IFI27, MAFA-AS1, and STC2 (**Figure 1A**). Gene Ontology (GO) enrichment analysis and chord diagrams demonstrated that these upregulated genes were significantly enriched in pathways related to the adaptive immune system (**Figures 1B, C**). Consistently, Kyoto Encyclopedia of Genes and Genomes (KEGG) pathway analysis revealed that these genes were predominantly involved in immune system-associated processes (**Figure 1D**). Subsequently, peripheral blood samples from an expanded cohort of 54 septic patients and 49 non-septic controls were analyzed (baseline characteristics are summarized in **Supplementary Table 2**). Clinical correlation analysis showed that IFI27 mRNA expression was positively associated with SOFA scores, APACHEII and HLA-DR expression, while exhibiting a negative correlation with PaO2/FiO2 ratio in septic patients (**Figures 2A–D**).

### IFI27 expression is increased in septic mice and lipopolysaccharide-treated BEAS-2B cells

**Figure 3:**
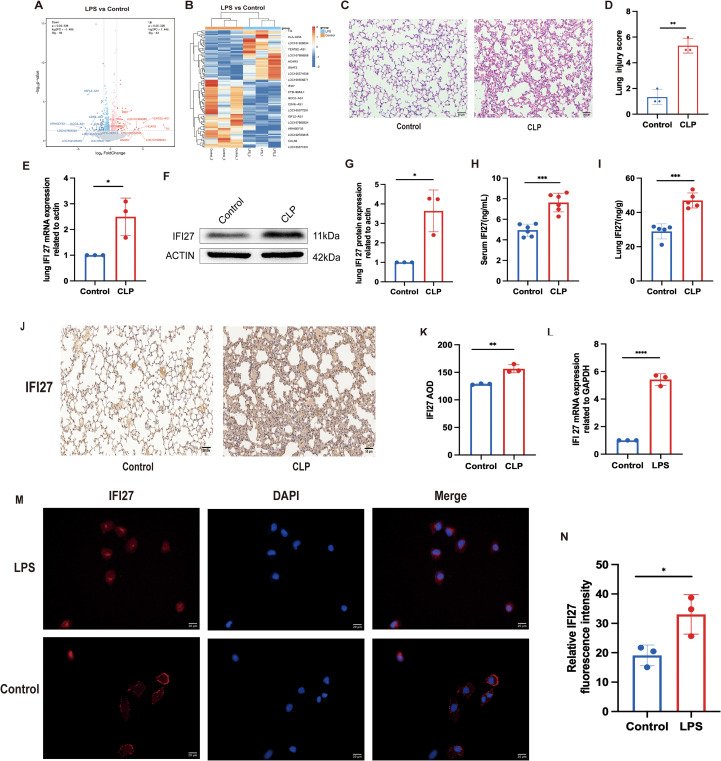
IFI27 expression level increased in septic mice and BEAS-2B cells. **(A, B)** Volcano plot of differentially expressed genes in BEAS-2 cells. The top ten upregulated genes were TG、IFI27、HLA−DRA、LOC101928234、YEATS2−AS1、LOC107986588、HCAR3、GNAT2、LOC105374538、LOC100506271. The top downregulated genes were CTB−99A3.1、GCC2−AS1、CDK6−AS1、LOC105377291、IGFL2−AS1、LOC107985524、ARHGEF33、LOC102723345、CALB2、LOC105371531. **(C)** Hematoxylin-eosin (H&E) staining image of mouse lung tissue. **(D)** Pathological score of mouse lung tissue was performed by smith lung injury score system. **(E)** RT-qPCR analysis of the relative mRNA expression level of IFI27. **(F-K)** Western blot、elisa analysis and immunohistochemistry analysis of the relative protein expression level of IFI27. **(L)** RT-qPCR and **(M, N)**immunofluorescence analysis of IFI27 expression level in BEAS-2B cells. p<0.05 indicated that the difference was statistically significant. Results were consistent across three independent experiments. *p < 0.05, **p < 0.01, ***p < 0.001, and ****p < 0.0001 by the one-way ANOVA followed Tukey’s multiple comparisons test. N = 3 for each group. P values less than 0.05 were considered statistically significant.

To investigate IFI27 expression *in vitro*, lung epithelial cells were treated with LPS, followed by total RNA extraction and transcriptomic sequencing. The top ten upregulated genes were identified as TG, IFI27, HLA-DRA, LOC101928234, YEATS2-AS1, LOC107986588, HCAR3, GNAT2, LOC105374538, and LOC100506271, with IFI27 showing marked upregulation in the LPS-treated group (**Figures 3A, B**). Histological analysis revealed significantly higher lung pathological injury scores in the CLP group compared with the control mice (**Figures 3C, D**). RT-qPCR analysis demonstrated elevated IFI27 transcript levels in lung tissues from septic mice (**Figure 3E**), which was further confirmed at the protein level by western blotting (**Figures 3F, G**). ELISA results showed increased IFI27 protein concentrations in both serum and lung tissues of septic mice (**Figures 3H, I**). Immunohistochemical staining further verified enhanced IFI27 expression in lung tissues (**Figures 3J, K**). *In vitro*, RT-qPCR and immunofluorescence analyses consistently demonstrated upregulated IFI27 expression in LPS-treated BEAS-2B cells (**Figures 3L–N**).

### IFI27 exacerbates sepsis-induced acute lung injury, and Treg depletion further aggravates tissue damage

**Figure 4:**
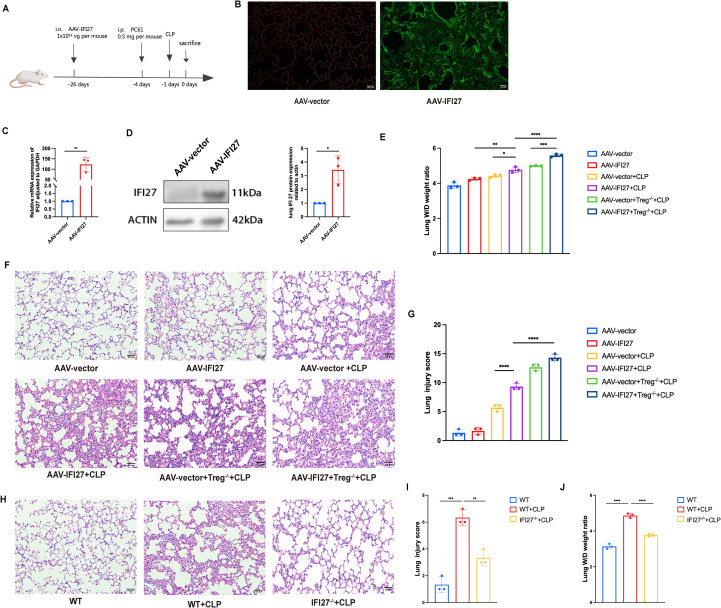
IFI27 exacerbated sepsis induced lung injury and depletion of Tregs further aggravated the injury. **(A)** WT mice and IFI27 overexpression mice were conducted, adeno-associated virus was utilized to transfect IFI27 overexpressed vector into mice via intranasal delivery. Three weeks later CLP modeling and the subsequent experiments were conducted thereafter. **(B)** lung tissues from AAV-IFI27-treated mice exhibited green fluorescence marking eGFP-tagged IFI27. **(C, D)** Expression of IFI27 increased in AAV-IFI27 group with RT-qPCR and western blot analysis. **(E)** Lung edema was measured by lung W/D weight ratio. **(F)** H& E staining of lung tissues from CLP-treated AAV-vector and AAV-IFI27 mice which followed Treg cell depletion (pc61 was injected 3 days before). **(G)** Lung injury scores of each group. **(H)** H& E staining of lung tissues from WT and IFI27^-/-^ group. **(I)** Lung injury scores of each group. **(J)** Lung edema was measured by lung W/D weight ratio. Results were consistent across three independent experiments. *p < 0.05, **p < 0.01, ***p < 0.001, and ****p < 0.0001 by the one-way ANOVA followed Tukey’s multiple comparisons test. N = 3 for each group. P values less than 0.05 were considered statistically significant.

**Figure 5:**
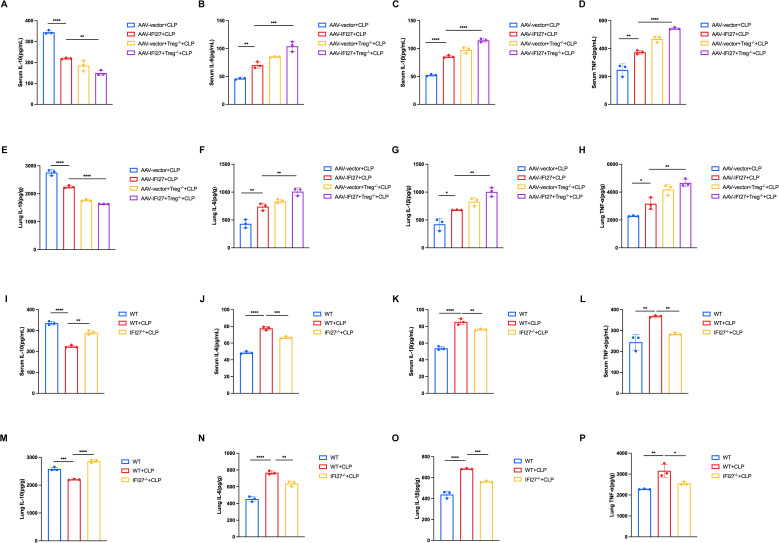
IFI27 exacerbated the inflammatory response and depletion of Tregs further aggravated the inflammation. **(A–P)** Expressions of mice serum and lung inflammatory cytokines (IL-10, IL-6, IL-1β and TNF-α). Results were consistent across three independent experiments. *p<0.05, **p<0.01, ***p<0.001, and****p<0.0001 by the one-way ANOVA followed Tukey’s multiple comparisons test. N = 3 for each group. P values less than 0.05 were considered statistically significant.

An IFI27 overexpression mouse model was generated using AAV-9 delivery **Figure 4A**). Fluorescence imaging of frozen lung sections revealed strong green fluorescence in the AAV-IFI27 group, confirming successful viral transduction of lung tissue (**Figure 4B**). RT-qPCR and western blot analyses showed significantly increased IFI27 expression in lung tissues of AAV-IFI27 mice compared with vector controls (**Figures 4C, D**). Functional assessment demonstrated that IFI27 overexpression significantly increased the lung W/D ratio, indicating aggravated pulmonary edema (**Figure 4E**). Histological evaluation further showed that IFI27 overexpression markedly exacerbated sepsis-induced lung injury (**Figures 4F, G**). The generation of IFI27 knockout mice and the genotyping results confirming homozygosity were provided in **Supplementary Figure 2**. A detailed longitudinal assessment of body weight and survival under these experimental conditions will be conducted in future studies. Histological evaluation further demonstrated that IFI27 knockout alleviated sepsis-induced lung injury (**Figures 4H, I**). IFI27 knockout Significantly reduced the lung W/D ratio, indicating relieved pulmonary edema (**Figure 4J**). ELISA analyses revealed elevated levels of pro-inflammatory cytokines (IL-6, IL-1β and TNF-α) in both serum and lung tissue, accompanied by a reduction in the anti-inflammatory cytokine IL-10 (**Figures 5A–H**) in IFI27-overexpressing CLP group. Furthermore, ELISA analyses revealed reduced levels of the pro-inflammatory cytokines IL-6, IL-1β, and TNF-α, accompanied by an increased level of the anti-inflammatory cytokine IL-10, in both the serum and lung tissue of the IFI27-knockout CLP group (**Figures 5I–P**). Following depletion of Tregs using a PC61 monoclonal antibody, lung injury was further aggravated in IFI27-overexpressing CLP mice, with enhanced pro-inflammatory cytokine production and further suppression of IL-10 expression (**Figures 4E–G**; **Figures 5A–H**).

### IFI27 promotes ferroptosis in pulmonary epithelial cells, which is further exacerbated by Treg depletion

**Figure 6:**
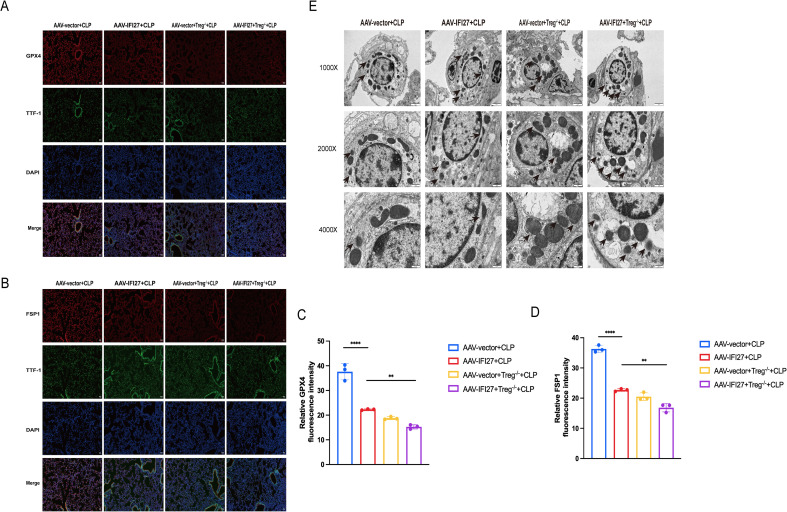
IFI27 exacerbated ferroptosis of pulmonary epithelial cells and depletion of Tregs further aggravated the injury. **(A, B)** Representative GPX4 and FSP1 fluorescence images of mouse lung epithelial cells. Scale bar, 50μm. **(C, D)** Relative GPX4 and FSP1 fluorescence intensity in each group. **(E)** Transmission electron microscopy (TEM) of ferroptosis in mouse lung epithelial cells. Results were consistent across three independent experiments. Data are expressed as mean ± standard error of the mean (SEM) (n = 3 for each group). **p < 0.01, ****p < 0.0001 by the one-way ANOVA followed Tukey’s multiple comparisons test. P values less than 0.05 were considered statistically significant.

To evaluate ferroptosis in alveolar epithelial cells, double immunofluorescence staining was performed using TTF-1 as a marker of alveolar epithelial cells. GPX4 and FSP1 are two key factors that regulate ferroptosis. The expression of the ferroptosis-related proteins GPX4 and FSP1 was significantly reduced in alveolar epithelial cells of IFI27-overexpressing CLP mice and further decreased following Treg depletion (**Figures 6A–D**). This indicates that IFI27 suppresses the expression of GPX4 and FSP1. To further explore whether IFI27 influences ferroptosis in epithelial cells by regulating Tregs, we depleted Tregs in mice and found that GPX4 and FSP1 expression decreased following Treg depletion. Thus, we hypothesize that IFI27 inhibits Tregs, thereby exacerbating ferroptosis in epithelial cells. To further observe the morphological changes associated with ferroptosis in lung epithelial cells, we examined mitochondrial morphology in epithelial cells using transmission electron microscopy. Transmission electron microscopy revealed more severe mitochondrial damage in type II alveolar epithelial cells from IFI27-overexpressing CLP mice, characterized by mitochondrial shrinkage, loss of cristae, and rupture of the outer mitochondrial membrane. These ultrastructural changes were further exacerbated upon Treg depletion (**Figure 6E**). The transmission electron microscopy results also indicate that IFI27 overexpression exacerbates ferroptosis in lung epithelial cells, in which Tregs play a critical role.

### IFI27 inhibits STAT5 phosphorylation and reduces Treg abundance and IL-10 secretion

**Figure 7:**
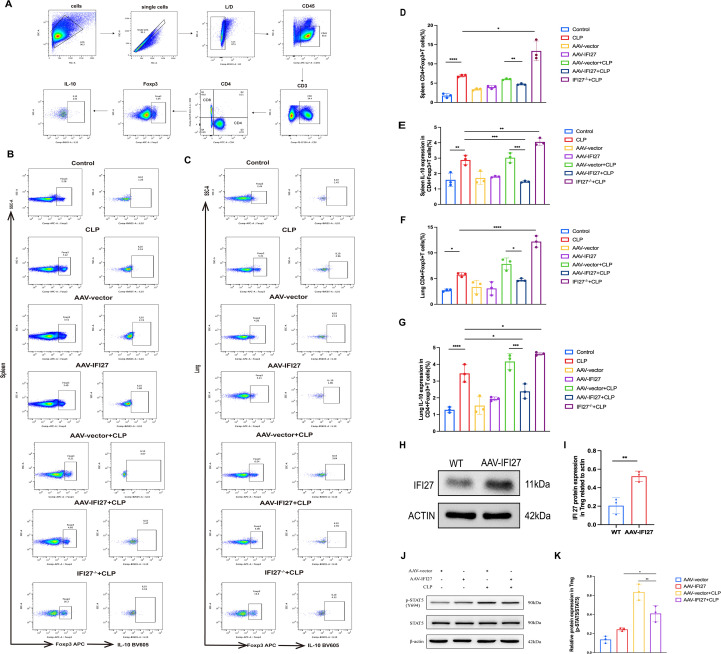
IFI27 inhibited p-STAT5 to decrease Treg cells and their secretion of IL-10. **(A)** Flowcytometry gate diagram of Treg cells and IL-10. **(B-G)** A representative flow cytometry result of mouse spleen and lung Treg cells were stained with foxp3 APC and IL-10 secreted by Treg cells were stained with BV605. **(H, I)** Western blot analysis of IFI27 protein expression in mice primary Treg cells of each group. **(J, K)** Western blot analysis of p-stat5 protein expression in mice primary Treg cells of each group. Results were consistent across three independent experiments. Data are expressed as mean ± SEM (n = 3 for each group). *p < 0.05, **p < 0.01, ***p < 0.001, and ****p < 0.0001 by the one-way ANOVA followed Tukey’s multiple comparisons test. P values less than 0.05 were considered statistically significant.

Flow cytometry was used to assess the regulatory effects of IFI27 on Tregs and on IL-10 secretion. A gating strategy was established to identify Tregs and intracellular IL-10 expression (**Figure 7A**). Compared with control mice, CLP mice exhibited increased proportions of Tregs and elevated IL-10 secretion in the spleen. In contrast, IFI27-overexpressing CLP mice showed a significant reduction in Treg frequency and IL-10 production compared with vector-treated CLP mice. IFI27 knockout CLP mice exhibited a significant increase in both Treg frequency and IL-10 production compared to WT CLP mice (**Figures 7B, D, E**). Similar trends were observed in lung tissues (**Figures 7C, F, G**). To further investigate how IFI27 influences Tregs, primary Tregs were isolated from mouse lung tissues, and protein lysates were subjected to Western blot analysis. IFI27 expression was detected in Tregs and was significantly elevated in the AAV-IFI27 group (**Figures 7H, I**), confirming IFI27 expression in Tregs. Subsequently, AAV-mediated IFI27 overexpression was employed to explore the underlying mechanisms by which IFI27 modulates Treg secretion. Western blot analysis demonstrated that IFI27 overexpression markedly reduced STAT5 phosphorylation in Tregs (**Figures 7J, K**). These findings suggest that IFI27 may suppress STAT5 phosphorylation, thereby diminishing Treg numbers and inhibiting IL-10 secretion by Tregs.

### IFI27-amplified Treg cells increased lipid peroxidation by suppressing IL-10/STAT3 signaling

**Figure 8:**
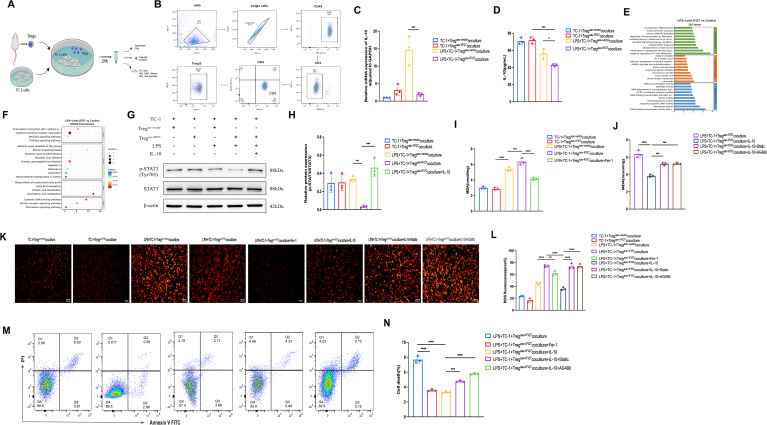
Amplified IFI27 in Treg cells contributed to increased lipid peroxidation by inhibiting IL-10/STAT3 signaling pathway. **(A)** Coculture of primary Tregs with TC-1 cells. **(B)** A representative flow cytometry result of the extraction efficiency of primary Treg cells in mice. **(C)** RT-qPCR analysis of the mRNA expression levels of IL-10 in Treg cells of the co-culture system. **(D)** Elisa analysis of the protein expression levels of IL-10 in Treg cells of the co-culture system. **(E)** Enriched gene ontology (GO) function analysis and Chord diagram of upregulated genes in BEAS-2B cells. Upregulated genes in BEAS-2B cells of LPS+Lenti-IFI27 groups were enriched in JAK-STAT signaling pathways, T cell differentiation and etc. **(F)** Kyoto Encyclopedia of Genes and Genomes (KEGG) enrichment analysis and Chord diagram of upregulated genes in BEAS-2B cells. Upregulated genes in BEAS-2B cells of LPS+Lenti-IFI27 groups were enriched in immune system, infectious disease, signal transduction and JAK-STAT signaling pathways. **(G, H)** Western blot analysis of p-stat3 protein expression in TC-1 cells of each group. **(I, J)** Lipid peroxidation levels in TC-1 cells were measured by MDA assay. **(K)** Representative ROS fluorescence images of TC-1 cells of the co-culture system. Scale bar, 100μm. **(L)** Relative ROS fluorescence intensity in each group. **(M)** Cell death determined by flow cytometry in TC-1 cells of the co-culture system. **(N)** Quantification of TC-1 cells death rate. Results were consistent across three independent experiments. Data are expressed as mean ± SEM (n = 3 for each group). *p < 0.05, **p < 0.01, ***p < 0.001, and ****p < 0.0001 by the one-way ANOVA followed Tukey’s multiple comparisons test. P values less than 0.05 were considered statistically significant.

To investigate the mechanistic role of IFI27-amplified Treg cells in epithelial injury, primary Tregs were isolated and co-cultured with mouse lung epithelial cells (**Figure 8A**). Flow cytometric analysis confirmed a Treg purity of 99.7% following isolation (**Figure 8B**). RT-qPCR and ELISA analyses showed that IFI27-amplified Tregs exhibited significantly reduced IL-10 expression and secretion compared with control Tregs under LPS stimulation (**Figures 8C, D**). These results indicate that Tregs with IFI27 overexpression inhibit IL-10 expression in the co-culture system. Transcriptomic sequencing of IFI27-overexpressing lung epithelial cells revealed enrichment of differentially expressed genes in JAK–STAT signaling and T-cell differentiation pathways, as demonstrated by GO and KEGG analyses (**Figures 8E, F**). Western blot analysis showed reduced STAT3 phosphorylation in lung epithelial cells co-cultured with IFI27-amplified Tregs, an effect that was reversed by exogenous IL-10 supplementation (**Figures 8G, H**). These findings indicate that IL-10 activates STAT3 phosphorylation in lung epithelial cells. Functional assays demonstrated increased ROS and MDA levels in epithelial cells co-cultured with IFI27-overexpressing Tregs, while IL-10 treatment significantly attenuated lipid peroxidation. These functional experiments suggest that IFI27-overexpressing Tregs exacerbate lipid peroxidation in lung epithelial cells, with IL-10 serving as a key regulatory factor in this process. To further investigate the effect of IFI27-overexpressing Tregs on ferroptosis in epithelial cells, we performed rescue experiments using the ferroptosis inhibitor Ferrostatin-1. Decreased ROS, MDA levels and mortality in epithelial cells were observed in LPS+TC-1+Tregaav-IFI27coculture+Fer-1 group. The results showed that the levels of ROS/MDA and mortality were specifically reversed in the Ferrostatin-1 group (**Figures 8I, K-N**). To explore the downstream mechanisms by which IL-10 induces ferroptosis in epithelial cells, we conducted additional rescue experiments using the STAT3 inhibitors Static and AG490. The results demonstrated that inhibition of STAT3 signaling with AG490 reversed the protective effects of IL-10 (**Figures 8I–L**). Flow cytometric analysis further confirmed that IL-10 reduced epithelial cell death, whereas STAT3 inhibition (static, AG490) restored cell death levels (**Figures 8M, N**).

## Discussion

This study highlights the role of IFI27-mediated Treg regulation in sepsis-induced pathological pulmonary damage. Through transcriptomic sequencing, we demonstrated that IFI27 is significantly upregulated in septic patients. Clinical analyses further showed that IFI27 expression levels in sepsis were positively correlated with disease severity and were closely associated with inflammatory responses and immune dysregulation. Animal experiments indicated that IFI27 expression was increased in septic mice and that IFI27 exacerbated sepsis-related pathological lung injury by inhibiting STAT5 phosphorylation, reducing Treg abundance, and suppressing IL-10 secretion. In addition, cell co-culture experiments demonstrated that IFI27-amplified Tregs promoted lipid peroxidation by inhibiting IL-10/STAT3 activation, thereby enhancing ferroptosis in septic pulmonary epithelial cells. Collectively, these findings suggest that IFI27-mediated regulation of Tregs contributes to sepsis-induced pulmonary pathological damage, with the IL-10/STAT3 signaling pathway playing a central mechanistic role.

IFI27 is a canonical interferon-stimulated gene, and recent studies have reported that IFI27 is enriched in mitochondria (28). Bioinformatics analyses have suggested that IFI27 may serve as a potential diagnostic marker for sepsis-induced ARDS (19). Moreover, a recent study reported increased expression of IFI27 in antigen-specific T cells from patients with COVID-19 (29), indicating a role for IFI27 in immune regulation. Consistent with these reports, our sequencing results revealed significant upregulation of IFI27 in septic patients. Furthermore, following lentiviral-mediated overexpression of IFI27 in BEAS-2B cells, transcriptomic analyses demonstrated that IFI27-associated genes were predominantly enriched in pathways related to T-cell differentiation, immune system processes, signal transduction, and JAK–STAT signaling. *In vivo*, we observed increased IFI27 expression at both mRNA and protein levels in serum and lung tissues of CLP mice, consistent with our sequencing data. Importantly, IFI27 overexpression in mice aggravated sepsis-induced lung injury and inflammatory responses, supporting a causal role for IFI27 in septic lung pathology.

Tregs are essential immune modulators that suppress excessive inflammatory responses through direct cell–cell interactions and the secretion of anti-inflammatory cytokines such as IL-10 and TGF-β (30). Inhibition of high mobility group box 1 (HMGB1) has been shown to increase Treg abundance and IL-35 expression, thereby alleviating caspase-11-mediated lung injury in CLP-induced acute lung injury models (31). Mechanistically, flow cytometry analysis demonstrated that IFI27 reduces both the number of Treg cells and the levels of IL-10 secretion in mouse spleen and lung tissues. Primary Treg cells were then isolated from mouse lung tissue, and the expression of IFI27 in these cells was confirmed by Western blotting. Following IFI27 gene amplification, both PCR and Western blot analyses further revealed upregulated IFI27 expression at the transcriptional and protein levels in Treg cells.

Given that STAT5 serves as a critical downstream effector of IL-2Rβ signaling and is essential for Treg development and maintenance (32), we next examined STAT5 expression in Treg cells by Western blotting. The results showed that STAT5 phosphorylation was suppressed after IFI27 gene amplification. Together, these findings indicate that IFI27 impairs Treg numbers and reduces IL-10 secretion is related to Treg/STAT5 changes. However, it is still unclear how IFI27 regulates STAT5 phosphorylation. The precise molecular mechanisms by which IFI27 regulates STAT5 activation and suppresses Treg differentiation remain to be fully elucidated.

The mechanisms underlying sepsis-induced pathological lung injury are complex, involving immune-mediated damage to lung epithelial cells. Increasing evidence indicates that ferroptosis of lung epithelial cells contributes to the pathophysiology of sepsis-induced pathological lung injury (33). Nevertheless, whether Tregs participate in regulating ferroptosis in lung epithelial cells has not been previously reported. Treg cells produce anti-inflammatory cytokines, such as IL-10 and TGF-β, which help suppress excessive inflammatory responses, alleviate lung tissue damage, and protect lung epithelial cells from inflammatory insult (13, 14). Studies have shown that IL-10 can inhibit ferroptosis in progenitor cells by activating the STAT3 signaling pathway to reduce lipid peroxidation (34). Our previous flow cytometry experiments revealed that IFI27 affects IL-10 secretion by Treg cells. Additionally, mRNA sequencing following IFI27 overexpression showed that the JAK-STAT signaling pathway was suppressed (**Figure 8F**). Therefore, we hypothesize that IFI27 regulates IL-10 secretion by Treg cells, and that IL-10 in turn modulates ferroptosis through the STAT3 signaling pathway. This led us to focus our mechanistic investigation on ferroptosis. The role of Treg cell-derived IL-10 in regulating ferroptosis in lung epithelial cells remains to be explored, offering a promising direction for theoretical innovation. In this study, we demonstrated that IFI27-amplified Tregs enhance ferroptosis in lung epithelial cells by reducing IL-10 production, providing novel insights into immune–epithelial crosstalk during sepsis.

STAT3 is a direct downstream effector of IL-10 signaling and is activated upon binding of IL-10 to its receptor (35). STAT3 phosphorylation promotes dimerization and nuclear translocation, thereby regulating target gene transcription (36). Mutations in IL-10 or its receptor IL-10RA impair STAT3 activation and lead to severe auto-inflammatory conditions, such as colitis with tissue damage and inflammatory infiltration, in both mice and humans (37).Consistent with these observations, our data demonstrate that IL-10 increases STAT3 Tyr705 phosphorylation in lung epithelial cells, indicating that the IL-10/STAT3 axis is involved in regulating epithelial cell function. Previous studies have shown that STAT3 overexpression exacerbates ROS accumulation and ferroptosis in osteosarcoma cells via regulation of the Nrf2/GPX4 axis (38), whereas increased STAT3 phosphorylation can alleviate ferroptosis by regulating SLC7A11 in mouse lung epithelial cells (39). In line with these findings, our results confirmed that inhibition of STAT3 activation in lung epithelial cells increased ROS and lipid peroxidation, thereby promoting ferroptosis. Together, these data indicate that the IL-10/STAT3 signaling pathway is a critical mediator of ferroptosis in lung epithelial cells. Our data strongly suggest that IFI27 exerts its effects via the IL-10/STAT3 axis in Treg cells, further studies (such as those using IL-10 neutralizing antibodies or conditional knockout mice) are warranted to confirm this pathway definitively.

**Figure 9:**
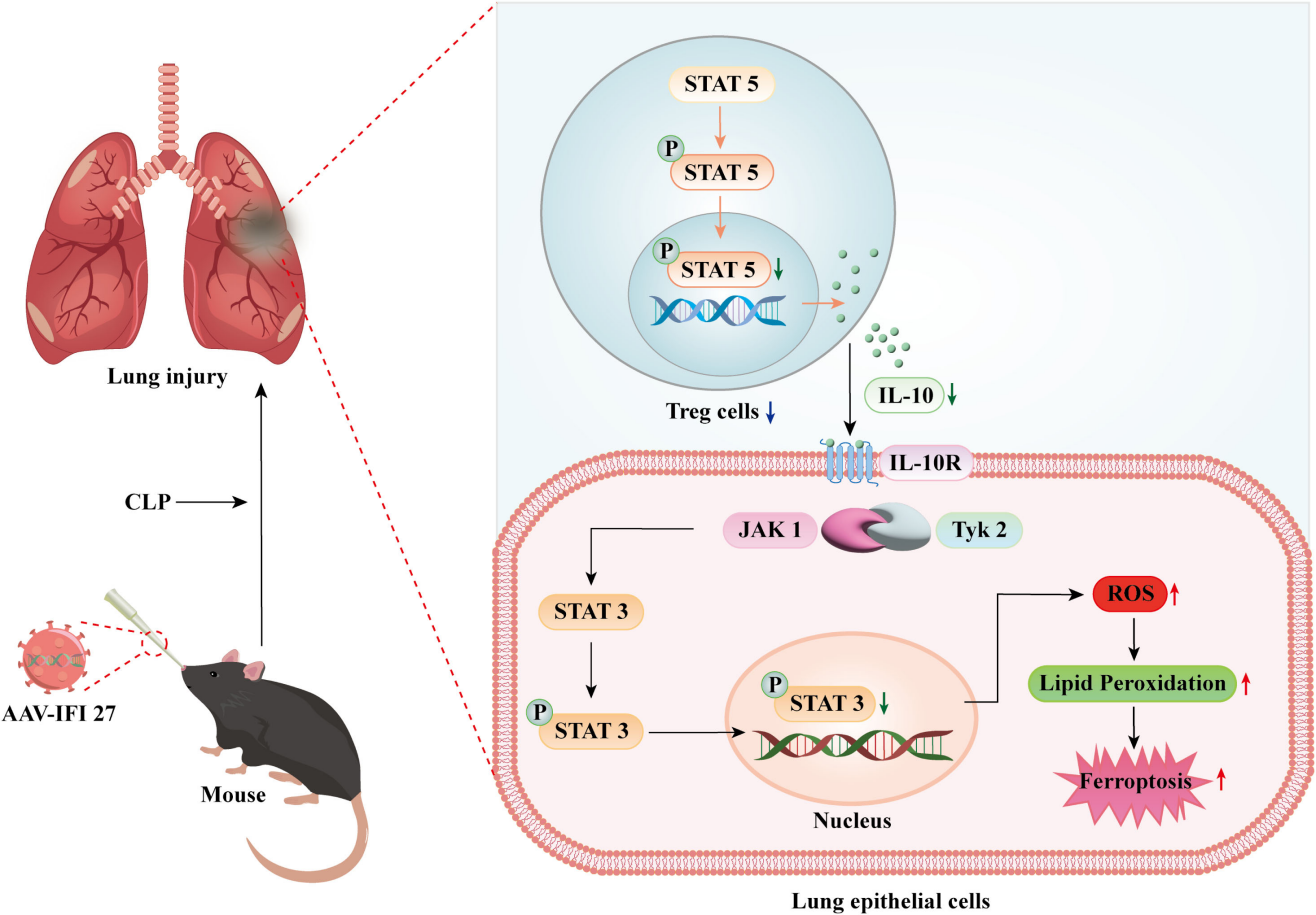
IFI27 acts as a key mediator in the pathogenesis of sepsis induced acute respiratory distress syndrome. Illustrative diagram demonstrates that the IFI27 regulates Treg cell suppression of IL-10/STAT3 pathway activation, leading to increased lipid peroxidation in septic lung epithelial cells, thereby enhancing ferroptosis and exacerbating sepsis-induced lung injury.

In summary, our study demonstrates that IFI27 exacerbates sepsis-induced ARDS by regulating Tregs to suppress activation of the IL-10/STAT3 signaling pathway, leading to increased lipid peroxidation and ferroptosis in lung epithelial cells (**Figure 9**).

## Data Availability

The datasets presented in this study can be found in online repositories. The names of the repository/repositories and accession number(s) can be found below: https://www.ncbi.nlm.nih.gov/, PRJNA1371076 PRJNA1371019.
