# Supplementary material for: IFI27-mediated regulation of regulatory T cells aggravates lung injury in sepsis via IL-10/STAT3 signaling
Source: Front Immunol. 2026 Apr 22;17:1760728. doi: 10.3389/fimmu.2026.1760728 (PMC13143761; doi:10.3389/fimmu.2026.1760728)
Supplement: Supplementary file 2 [file DataSheet2.docx]

Supplementary Table 1 The primer sequences for gene amplification

| Gene |  | Primer sequences |
| --- | --- | --- |
| IFI27 | Forward | 5'-ACTGGTCCTCATGGCGTTTT-3' |
|  | Reverse | 5'-CAGCTACTGTCATGGCTCCC-3 |
| GAPDH | Forward | 5'-AGGTCGGTGTGAACGGATTTG-3' |
|  | Reverse | 5'-TGTAGACCATGTAGTTGAGGTCA-3' |
| IL-10 | Forward | 5'-AAGGGTTACTTGGGTTGCCA-3' |
|  | Reverse | 5'-GCCTTGTAGACACCTTGGTCTT-3' |

Supplementary Table2 Baseline characteristics between septic patients and controls.

| Variables | Control  (n=49) | Sepsis  (n=26) | P value |
| --- | --- | --- | --- |
| Demographics |  |  |  |
| Age, median(IQR) | 68(64, 74) | 68(62, 76) | 0.85 |
| Sex,（male，n%) | 29(59.2) | 21(80.8) | 0.061 |
| Infection sources, (%) |  |  |  |
| Gastrointestinal tract |  | 9(34.6) |  |
| Urogenital tract |  | 14(53.8) |  |
| Skin soft tissue |  | 2(7.7) |  |
| Disease severity score |  |  |  |
| SOFA | 0(0,0) | 3(2,5) | <0.001 |
| APACHEII | 5(3,5) | 19(13,24) | <0.001 |
| Laboratory parameters |  |  |  |
| CRP, mg/L, median(IQR) | 2(1, 3) | 154(67, 175) | <0.001 |
| PCT, ng/mL, median(IQR) | 0.05(0.04, 0.08) | 7.71(4.68, 34.24) | <0.001 |
| WBC, *10^9^/L, median(IQR) | 6.22(5.1, 7.57) | 8.84(5.34, 13.44) | 0.002 |
| LYM, *10^9^/L, median(IQR) | 1.54(1.23, 1.98) | 0.89(0.52, 1.1) | <0.001 |
| Prealbumin, mg/L, median(IQR) | 242.5(194.3,265.8) | 131(96, 168) | <0.001 |
| IL-6, pg/mL, median(IQR) | 2.76(2.0, 4.89) | 22.2(11.25, 98.5) | <0.001 |
| IL-10, pg/mL, median(IQR) | 5(5, 5) | 5(5, 11.57) | <0.001 |
| IL-8, pg/mL, median(IQR) | 12.4(9.9, 20.4) | 18.9(14.7, 62.45) | <0.001 |
| TNF-α, pg/mL, median(IQR) | 10.2(6.9, 13.2) | 23.1(16.8, 40.9) | <0.001 |
| IL-2R, U/mL, median(IQR) | 329(243, 446) | 903(646, 2701) | <0.001 |
| CD3 cells/uL, median(IQR) |  | 541(311, 715) |  |
| CD4 cells/uL, median(IQR) |  | 280(205, 395) |  |
| CD8 cells/uL, median(IQR) |  | 189(105, 257) |  |
| CD16+CD56+cells/uL, median(IQR) |  | 122(57, 176) |  |
| CD19 cells/uL, median(IQR) |  | 88(58, 144) |  |
| HLA-DR, MFI, median(IQR) |  | 2420(1258, 3431) |  |
| Ventilation, n(%) |  | 2(7.69) |  |

Supplementary Table3 Baseline characteristics between septic patients and controls in the RNA sequencing groups.

| Variables | Control  (n=4) | Sepsis  (n=4) | P value |
| --- | --- | --- | --- |
| Demographics |  |  |  |
| Age, year(Mean ± SD) | 71.75±3.3 | 63.25±5.8 | 0.25 |
| Sex,（male，n%) | 3(75) | 3(75) | >0.05 |

Primary disease, n (%)

| Cardiovascular disease | 0 | 0 |  |
| --- | --- | --- | --- |
| Hypertension | 2 | 4 | 0.127 |
| Diabetes | 1 | 2 | 0.5 |
| COPD | 0 | 0 |  |
| Kidney disease | 0 | 3 | 0.05 |
| Chronic liver disease | 0 | 0 | <0.001 |
| Cance | 0 | 0 |  |
