# Supplementary material for: IFI27-mediated regulation of regulatory T cells aggravates lung injury in sepsis via IL-10/STAT3 signaling
Source: Front Immunol. 2026 Apr 22;17:1760728. doi: 10.3389/fimmu.2026.1760728 (PMC13143761; doi:10.3389/fimmu.2026.1760728)
Supplement: Supplementary file 3 [file DataSheet3.docx]

Supplementary Figure1 Anti-CD25 mAb (PC61) partially depletes Foxp3+ cells.


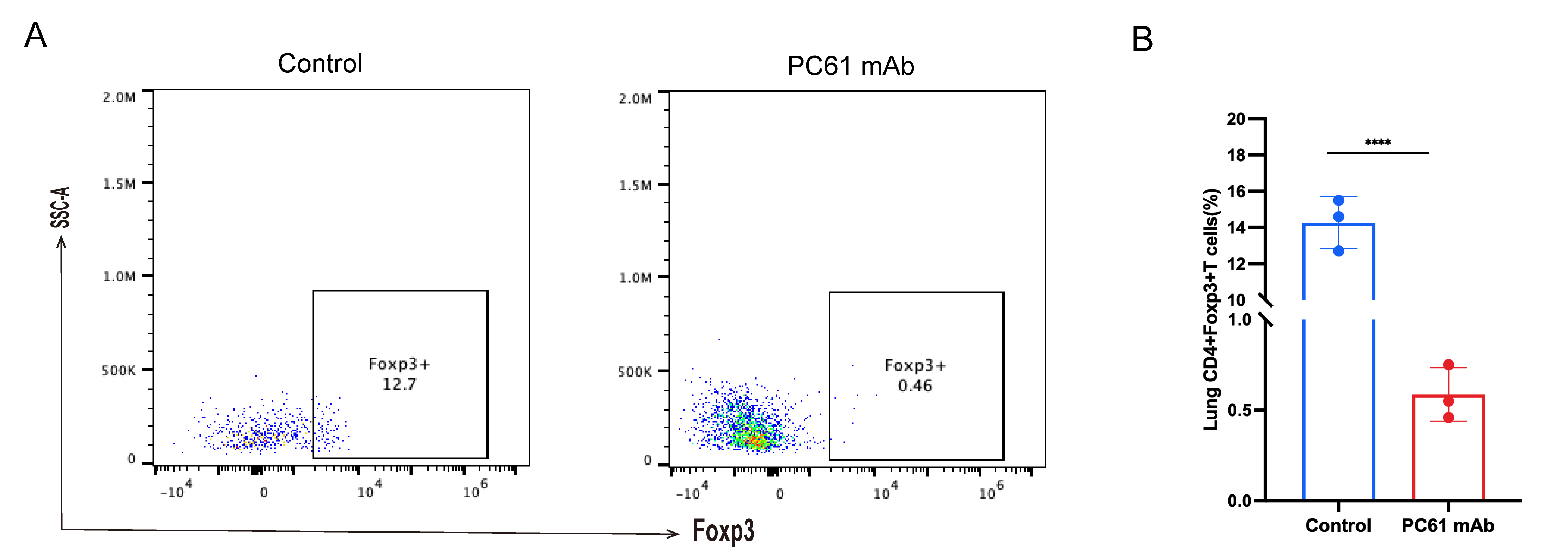


Anti-CD25 mAb (PC61) partially depletes Foxp3+ cells. (A) Flow cytometric profile of lung CD4+ T cells in untreated or PC61-treated mice. (B) There was significantly difference in the Treg level between untreated and PC61-injected mice (n=3). ****P < 0.0001.

Supplementary Figure2 Genotypic Identification of IFI27 knockout mice


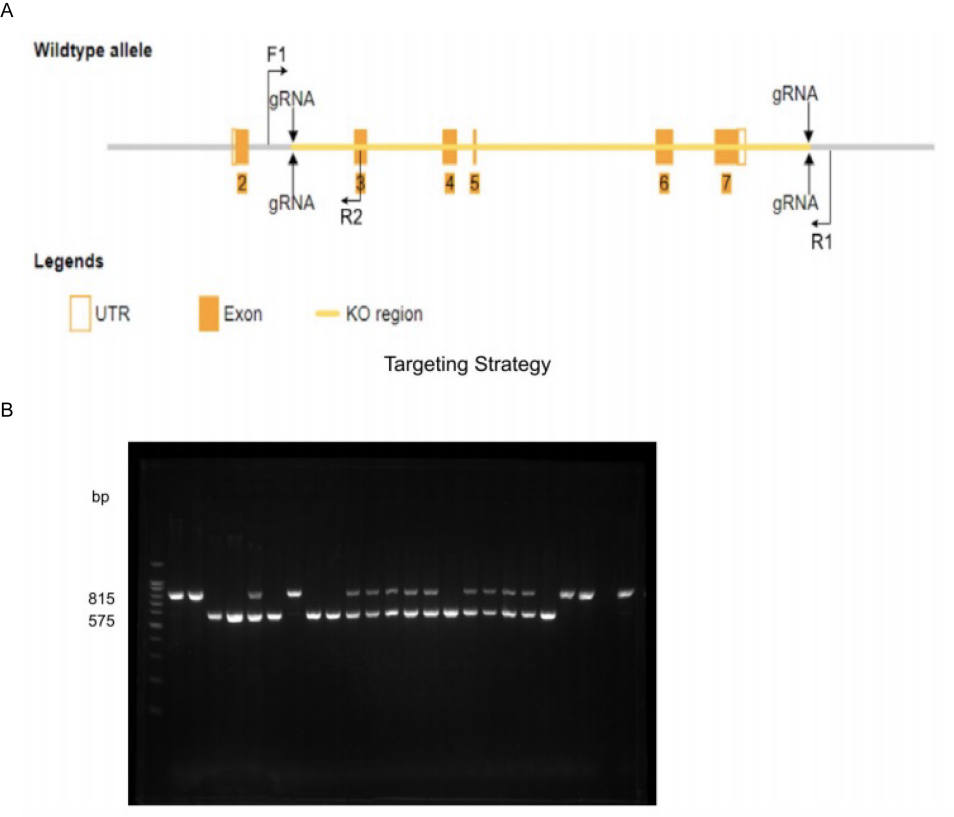


Genotyping of IFI27 knockout mice by DNA analysis: heterozygous mice show both bands, homozygous mice show a single band at 575 bp, and wild type mice show a single band at 815 bp.

Supplementary Figure3 Targeting Strategy of the IFI27 knockout mice


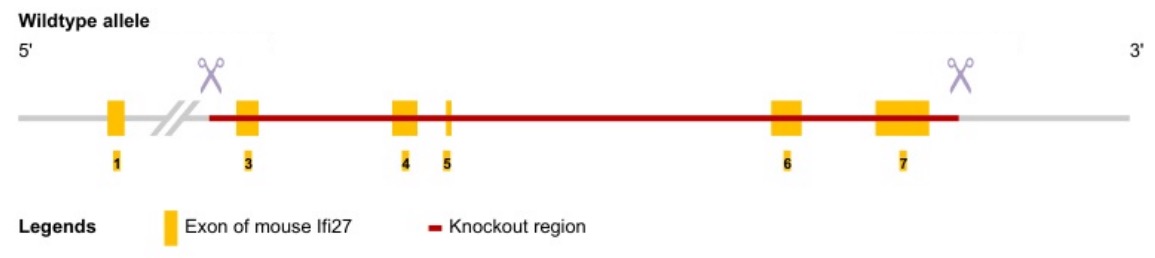


To create a Ifi27 knockout mouse model (C57BL/6JCya) using gene editing technology.The Ifi27 gene (NCBI Reference Sequence: NM_026790; Ensembl: ENSMUSG00000064215) is located on mouse chromosome 12. Seven exons are identified, with the ATG start codon in exon 2 and the TAA stop codon in exon 7 (Transcript Ifi27-205: ENSMUST00000085065). Exon 3~7 will be selected as target site. The region contains 665 bp coding sequence.This strategy is designed based on genetic information in existing databases.
